# Prevalence and Risk Factors for Oral HPV Infection in Young Australians

**DOI:** 10.1371/journal.pone.0091761

**Published:** 2014-03-17

**Authors:** Annika Antonsson, Michelle Cornford, Susan Perry, Marcia Davis, Michael P. Dunne, David C. Whiteman

**Affiliations:** 1 Department of Population Health, QIMR Berghofer Medical Research Institute, Brisbane, Australia; 2 School of Public Health and Social Work, Queensland University of Technology, Brisbane, Australia; Georgetown University, United States of America

## Abstract

The prevalence of human papillomavirus (HPV)–associated head and neck cancers is increasing, but the prevalence of oral HPV infection in the wider community remains unknown. We sought to determine the prevalence of, and identify risk factors for, oral HPV infection in a sample of young, healthy Australians. For this study, we recruited 307 Australian university students (18–35 years). Participants reported anonymously about basic characteristics, sexual behaviour, and alcohol, tobacco and illicit drugs use. We collected oral rinse samples from all participants for HPV testing and typing. Seven of 307 (2.3%) students tested positive for oral HPV infection (3 HPV-18, one each of HPV-16, -67, -69, -90), and six of them were males (p = 0.008). Compared to HPV negative students, those with oral HPV infection were more likely to have received oral sex from more partners in their lifetime (p = 0.0004) and in the last year (p = 0.008). We found no statistically significant associations with alcohol consumption, smoking or numbers of partners for passionate kissing or sexual intercourse. In conclusion, oral HPV infection was associated with male gender and receiving oral sex in our sample of young Australians.

## Introduction

The incidence of squamous cell carcinoma of the head and neck (HNSCC), more specifically of oropharyngeal squamous cell carcinoma (OPSCC), is increasing. Sites particularly affected are those of the tonsils and base of tongue [1]–[3]. While previously most of these cancers could be attributed to tobacco and alcohol, it appears that recently fewer patients have been presenting with these classical risk factors. During the last decade there has been a sizeable increase in the numbers of OPSCCs associated with human papillomavirus (HPV), and these patients are more often non-smokers, and often somewhat younger than those with HPV negative OPSCC [2]–[7]. This is believed to reflect an increasing prevalence of oral HPV infections due to changing sexual practices [4], [5]. HPV-16 is the most common high-risk HPV type identified in oral samples from the general population as well as in HNSCC tumours [5], [8]–[10]. Oral HPV infection has been strongly implicated as being responsible for the increase in HNSCC and is the likely causative factor of the increasing subset of HNSCC among young non-smokers, but very little is known about the epidemiology of oral HPV infections. Previous studies have shown varying oral α-HPV prevalence in different populations, ranging from 0.6% (4/662) in a Japanese study of a sample of the population of Miyako Island up to 25% (29/117) in an American study of older men [10]–[14]. The largest study to date reported an overall oral HPV prevalence of 6.9% among 5,579 men and women, aged 14 to 69 years in the United States [10]. The prevalence of oral infection in the general population of Australia remains unknown. We analysed α-HPV DNA prevalence and HPV types together with life-style factors and sexual behaviour in a sample of young, healthy Australians, and herewith report the findings.

## Materials and Methods

### Study population and samples

We recruited 307 students (ages 18 to 35 yrs) from an Australian university. Students were informed about our study by emailing flyers, posting on social media through student organisations and providing information in lectures. Students were approached after lectures as well as at campus where our data collection room was set up. Participants were asked to self-complete a paper-based 4-page questionnaire. Potential participants were informed that the investigators would not record any identifying details, and that their anonymity would be preserved. Students who agreed to participate were given a questionnaire and a tube for collection of oral rinse sample, both labelled with the same unique identification number. The questionnaire covered basic demographics (age, gender, education etc), sexual behaviour (passionate kissing, giving and receiving oral sex, and sexual intercourse, numbers of partners, recency of various sexual activities and sexual orientation), and alcohol, tobacco and illicit drugs use as well as history of genital infections, abnormal pap smears and Gardasil vaccination (the quadrivalent HPV vaccine that protects against HPV types HPV-6, -11, -16 and -18 and for which Australia launched a national program in 2007). It also included two questions about knowledge of protection from contracting a sexually transmitted infection from engaging in oral sex. In the questionnaire we included the following descriptions: ‘one drink’  =  a glass of wine, middy of beer or nip of spirits; ‘Non-smoker’ - You have smoked 100 or fewer cigarettes in your life-time; An ‘illicit drug’ refers to illegal or controlled substances; ‘Recreational drug’ refers to drugs used for non-medical purposes, not including cigarettes or alcohol; ‘Passionate kissing’ refers to open-mouthed kissing; ‘Oral sex’ refers to a man's or woman's mouth on a partner's genital area; ‘Sexual intercourse’ refers to vaginal intercourse and anal intercourse only and does not refer to oral sex; and ‘Mouth jewellery’ refers to jewellery worn on the lips or tongue requiring piercing of the skin.

Participants rinsed their mouth with 7 ml sterile saline (0.9%) for 30 seconds. All oral rinse specimens were frozen at −20°C within 3 hours of collection. Specimens were later thawed at ambient room temperature, vortexed and 3 ml was used for DNA extraction. DNA was extracted in batches of 24 samples at a time, using the QIAamp DNA Mini Kit following the QIAGEN supplementary protocol for Isolation of genomic DNA from saliva and mouthwash (QA19v Jul-10).

### Ethics Statement

All participants were given a copy of the Study Information Sheet; consent was obtained by completing the anonymous questionnaire. The study was approved by the human research ethics committees of the Queensland University of Technology (1200000292) and the QIMR Berghofer Medical Research Institute (P1508).

### HPV DNA analysis

The DNA samples extracted from the participants' oral rinse specimens were analysed by PCR for the presence of HPV with the general mucosal HPV primer GP5+/GP6+ [15]. β-globin PCR with the primers PCO3 and PCO4 [16] was carried out on all samples to ensure that no PCR inhibiting agents were present and that they contained human DNA. The final volume of PCR solution (25 μl) contained 5 μl of extracted sample DNA, 0.5 μM of the GP5+ and GP6+ primers or PCO3 and PCO4 primers (Sigma-Aldrich), dNTPs at concentrations of 0.2 mM each (Roche), 1 U of AmpliTaq Gold DNA polymerase, 1× PCR Gold buffer and 2.0 mM MgCl_2_ (Applied Biosystems). Forty cycles of amplification was performed on an Eppendorf Mastercycler Gradient PCR machine after an initial step of 10 minutes denaturation at 94°C. Each cycle for the GP+ PCR consisted of 94°C for 1 min, 40°C for 2 min and 72°C for 1.5 min, plus a final elongation step at 72°C for 4 min. Each amplification cycle for the β-globin PCR consisted of 94°C for 1.5 min, 50°C for 1.5 min and 72°C for 1.5 min, plus a final elongation step at 72°C for 4 min. In each batch of tests, H_2_O was used as a negative control. HeLa cells (HPV-18 positive cervical cell line) were used as a positive control in both PCR reactions. PCR amplicons were analysed by electrophoresis (1.5% agarose gel containing ethidium bromide; SeaKem, FMC bioproducts and Sigma) and identified under UV light. To minimize the risk for contamination we used different pipettes and rooms for DNA extraction, preparing the PCR solution, adding DNA samples to PCR solution and electrophoresis analysis.

### HPV Type Determination

The HPV positive PCR products were purified with the Agencourt® AMPure PCR purification kit (Agencourt Bioscience) in a magnetic 96-ring SPRIplate®. The sequencing reaction contained the purified PCR products together with 3.25 μM of primer and BigDye Terminator (Applied Biosystems). The sequencing reaction was performed in an Eppendorf Mastercycler Gradient PCR machine. After an initial step of 96°C for 2 min, 20 cycles followed, each with 96°C for 10 s, 50°C for 5 s and 60°C for 2 minutes. The sequence reactions were purified with the Agencourt® CleanSEQ dye-terminator removal kit (Agencourt Bioscience) in a magnetic 96-ring SPRIplate®, and purified sequence reactions were analysed with an automated DNA sequencer (ABI model 3100). The DNA sequences obtained were compared with available sequences in GenBank through the BLAST server (http://blast.ncbi.nlm.nih.gov).

### Statistical Analysis

We conducted a sample size calculation for this study, and concluded that a sample of 300 would be sufficient to detect a prevalence of 5% with 80% power (expected to observe 15 events, and would have 95% confidence that the true population prevalence would be between 2.5 and 7.5%).

We compared HPV positive with HPV negative individuals to identify characteristics associated with HPV positivity. For comparisons, we used the t-test for normally distributed continuous variables and for categorical variables we used the χ2 test (or Fischer's exact test when the expected number in any cell was <5). Odds ratios (ORs) and 95% confidence intervals (CIs) were obtained using logistic regression models. For some exposures, categories were combined to permit effects to be estimated. All significance tests were two-sided tests at α = 0.05. We used SAS (version 9.2) for all analyses.

## Results

### Characteristics of the study population

The mean age of the 307 participants was 22.0 years (range 18–35) and 62% were female. The majority of students (90%; n = 275) had had some type of sexual contact (including passionate kissing, oral sex and sexual intercourse) in their lifetime. Of the students who reported prior sexual activity, 82% (n = 225) had engaged in oral sex, and 78% (n = 214) had had sexual intercourse. Thirty-two percent of the participants (n = 98, 95 females, 3 males) had received at least one dose of the Gardasil® vaccine. Table 1 presents characteristics of the study population in detail.

**Table 1:** Selected characteristics of the 307 participants.

Characteristics		N (%)
Gender	Male	116 (37.8%)
	Female	191 (62.2%)
Age	18–19 yrs	98 (31.9%)
	20–21	80 (26.1%)
	22–24	66 (21.5%)
	25–35	63 (20.5%)
Born in Australia	No	83 (27.0%)
	Yes	224 (73.0%)
Number of alcoholic drinks per week	Less than 1	162 (52.8%)
	2 or more	145 (47.2%)
Smoking status	Current	19 (6.2%)
	Former	34 (11.1%)
	Non	254 (82.7%)
Illicit drug use in the last 12months	Never	219 (71.3%)
	Less than monthly	65 (21.2%)
	More than monthly	23 (7.5%)
Ever had any type of sexual contact (including passionate kissing)	No	31 (10.1%)
	Yes	276 (89.9%)
Ever kissed another person passionately	No	31 (10.1%)
	Yes	276 (89.9%)
Ever engaged in oral sex	No	51 (18.5%)
	Yes	224 (81.5%)
Gender of oral sex partner	Same sex only	20 (8.9%)
	Opposite sex only	193 (85.8%)
	Both sexes	12 (5.3%)
Ever given oral sex	No	9 (4.0%)
	Yes	216 (96.0%)
Use of condom when giving oral sex	Never	182 (83.9%)
	Seldom	15 (6.9%)
	Occasionally	16 (7.4%)
	Frequently	2 (0.9%)
	Very frequently	2 (0.9%)
Ever received oral sex	No	11 (4.9%)
	Yes	214 (95.1%)
Ever had sexual intercourse	No	62 (22.5%)
	Yes	214 (77.5%)
Gender of sexual intercourse partners	Same sex only	14 (6.5%)
	Opposite sex only	190 (88.4%)
	Both sexes	11 (5.1%)
Have you ever received the Gardasil vaccine	No	199 (65.0%)
	Yes	98 (32.0%)
	Don't know	9 (3.0%)

### HPV prevalence and HPV types

Seven of the 307 (prevalence 2.3%; 95% confidence interval 0.6–4.0%) students tested positive for oral HPV infection. All the positive samples had single HPV types detected. The most prevalent HPV type was HPV-18 which was identified in three of the seven oral HPV positive individuals (43%). HPV-16, -67, -69 and -90 were identified in one sample each. All samples tested positive for β-globin.

### Characteristics and behaviour associated with oral HPV infection

Table 2 shows characteristics and behaviour for HPV positive and HPV negative individuals in detail. Six of the seven oral HPV positive individuals were males (p = 0.008). The mean age of the oral HPV positive group was 23.0 (SD 1.6) and 22.0 (SD 4.0) for the HPV negative. One of the seven oral HPV positive individuals reported to have kissed passionately, but had never engaged in oral sex or had sexual intercourse. Students with oral HPV infection reported having received oral sex from more partners in their lifetime (p = 0.0004) as well as in the past year (p = 0.008) compared to HPV negative subjects. Self-reported diagnosis of HIV was significantly associated with oral HPV infection (p = 0.023). The following factors were not statistically significantly associated with oral HPV infection in this sample: age, county of birth, language spoken at home, secondary school education, alcohol consumption, illicit drug use, smoking, tonsillectomy, braces, mouth jewellery, age at first passionate kiss, giving/receiving oral sex or sexual intercourse, number of partners for passionate kissing, giving oral sex and sexual intercourse, partners of same, opposite or mixed sex, previous history of genital chlamydia, gonorrhoea, HPV or herpes simplex, and use of condom or dental dam while giving or receiving oral sex. None of the oral HPV positive subjects reported having had an abnormal pap smear or had a partner with an abnormal pap smear. None of the seven students with oral HPV infection had been vaccinated with Gardasil®.

**Table 2:** Characteristics of HPV negative and HPV positive individuals.

Characteristics		HPV-	HPV+	Crude OR	
		N (%)	N (%)	(95% CI)	P value
Gender	Male	110 (36.7%)	6 (85.7%)	1.00 (ref)	0.008
	Female	190 (63.3%)	1 (14.3%)	0.10 (0.01–0.81)	
Age	18–19 yrs	96 (32.0%)	2 (28.6%)	1.00 (ref)	
	20–21	79 (26.3%)	1 (14.3%)	0.61 (0.05–6.82)	
	22–24	65 (21.7%)	1 (14.3%)	0.74 (0.07–8.31)	
	25–35	60 (20.0%)	3 (42.9%)	2.40 (0.39–14.78)	0.508
Born in Australia	No	80 (26.7%)	3 (42.9%)	1.00 (ref)	0.340
	Yes	220 (73.3%)	4 (57.1%)	0.48 (0.11–2.21)	
Number of alcoholic drinks per week	Less than 1	159 (53.0%)	3 (42.9%)	1.00 (ref)	0.595
	2 or more	141 (47.0%)	4 (57.1%)	1.50 (0.33–6.83)	
Smoking status	Non	250 (83.3%)	4 (57.1%)	1.00 (ref)	0.191
	Current	18 (6.0%)	1 (14.3%)	3.47 (0.37–32.71)	
	Former	32 (10.7%)	2 (28.6%)	3.91 (0.69–22.18)	
Illicit drug use in the last 12months	Never	216 (72.0%)	3 (42.9%)	1.00 (ref)	0.072
	Less than monthly	63 (21.0%)	2 (28.6%)	2.29 (0.37–13.98)	
	More than monthly	21 (7.0%)	2 (28.6%)	6.86 (0.37–13.98)	
Ever had any type of sexual contact (including passionate kissing)	No	31 (10.3%)	0 (0.0%)	–	0.370
	Yes	269 (89.7%)	7 (100%)	–	
Number of kissing partners in a lifetime	1	21 (7.9%)	1 (14.3%)	1.00 (ref)	0.504
	2–3	55 (20.6%)	1 (14.3%)	0.38 (0.02–6.39)	
	4–7	59 (22.1%)	0 (0.0%)	–	
	8–15	52 (19.5%)	1 (14.3%)	0.40 (0.02–6.76)	
	16–28	31 (11.6%)	1 (14.3%)	0.68 (0.04–11.44)	
	29 or more	49 (18.4%)	3 (42.9%)	1.29 (0.13–13.09)	
Ever engaged in oral sex	No	50 (18.7%)	1 (14.3%)	1.00 (ref)	0.769
	Yes	218 (81.3%)	6 (85.7%)	1.38 (0.16–11.69)	
Gender of oral sex partner	Same sex only	19 (8.7%)	1 (16.7%)	1.00 (ref)	
	Opposite sex only	188 (85.8%)	5 (83.3%)	0.50 (0.06–4.55)	
	Both sexes	12 (5.5%)	0 (0.0%)	-	0.686
Ever given oral sex	No	9 (4.1%)	0 (0.0%)	-	0.612
	Yes	210 (95.9%)	6 (100%)	-	
Number of partners given oral sex to in lifetime	1–7	169 (80.5%)	3 (50.0%)	1.00 (ref)	0.068
	8 or more	31 (19.5%)	3 (50.0%)	4.12 (0.80–21.17)	
Number of partners given oral sex to in last year	None	22 (10.4%)	1 (16.7%)	1.00 (ref)	0.262
	1	130 (61.6%)	2 (33.3%)	0.34 (0.03–3.89)	
	2–3	38 (18.0%)	1 (16.7%)	0.58 (0.03–9.72)	
	4 or more	21 (10.0%)	2 (33.3%)	2.10 (0.18–24.87)	
Use of condom when giving oral sex	Never	176 (83.4%)	6 (100%)	-	0.880
	Seldom	15 (7.1%)	0 (0.0%)	-	
	Occasionally	16 (7.6%)	0 (0.0%)	-	
	Frequently	2 (0.9%)	0 (0.0%)	-	
	Very frequently	2 (0.9%)	0 (0.0%)	-	
Ever received oral sex	No	10 (4.6%)	1 (16.7%)	1.00 (ref)	0.175
	Yes	209 (95.4%)	5 (83.3%)	0.24 (0.03–2.24)	
Number of partners received oral sex from in lifetime	1	55 (25.8%)	0 (0.0%)	-	0.0004
	2–3	66 (31.0%)	0 (0.0%)	-	
	4–7	50 (23.5%)	1 (20.0%)	1.00 (ref)	
	8–15	29 (13.6%)	1 (20.0%)	1.72 (0.10–28.62)	
	16–28	8 (3.8%)	2 (40.0%)	12.50 (1.01–154.40)	
	29 or more	5 (2.3%)	1 (20.0%)	10.00 (0.54–185.46)	
Number of partners received oral sex from last year	None	25 (11.7%)	1 (20.0%)	1.00 (ref)	0.008
	1	133 (62.4%)	1 (20.0%)	0.19 (0.01–3.10)	
	2–3	31 (14.6%)	0 (0.0%)	-	
	4 or more	24 (11.3%)	3 (60.0%)	3.12 (0.30–32.16)	
Ever had sexual intercourse	No	61 (22.7%)	1 (14.3%)	1.00 (ref)	0.599
	Yes	208 (77.3%)	6 (85.7%)	1.76 (0.21–14.90)	
	No	300 (100%)	6 (85.7%)	-	
Ever diagnosed with or treated for HIV/AIDS	Yes	0 (0.0%)	1 (14.3%)	-	
	No	300 (100%)	6 (85.7%)	-	0.023
Have you ever received the Gardasil vaccine	No	192 (64.2%)	7 (100%)	-	0.146
	Yes	98 (32.8%)	0 (0.0%)	-	
	Don't know	9 (3.0%)	0 (0.0%)	-	

We asked if the participants thought it was possible to get HPV from engaging in oral sex and 58% answered yes, 3% no, 8% not impossible, but rare, and 31% were not sure. We also investigated the knowledge of protection from STIs when engaging in oral sex and the majority of students (91%) thought that use of male condom would be protective. Table 3 presents the results of all means of protection that were included in the questionnaire.

**Table 3:** Proportion of positive responses to nominated measures against STI when engaging in oral sex.

Means of protection	yes n (%)
Male condom	279 (91)
Female condom	126 (41)
Cling wrap	15 (5)
Spermicide	5 (2)
Oral sex, not involving ejaculation	18 (6)
Contraceptive patch	5 (2)
Dental dam	72 (23)
Contraceptive pill	7 (2)
Intrauterine device	3 (1)

## Discussion

Here we investigated the prevalence and determinants of oral HPV infections in a sample of young Australians. We obtained DNA samples from oral rinse samples and used PCR primers (GP+) to detect a wide spectrum of α-papillomaviruses [15], and found an oral HPV prevalence of 2.3% in our sample. Previous reports of oral HPV prevalence have varied greatly between different geographic areas. The largest study on oral HPV prevalence in the United States, performed by Gillison *et al* on 5,579 individuals, reported an overall prevalence of 6.9% [10]. In our study the age group 18–24 years had an oral HPV prevalence of 1.6% (4/244) and the US study 5.6%, and the age group 25–35 years 5.0% (3/60) compared to 7.1% and 7.3% for the US study's age groups 25–29 years and 30–34 years, respectively [10]. The oral HPV prevalence was lower in our study, but both studies showed an increasing prevalence with age. The oral α-HPV prevalences in several American studies have ranged from 3.3% to 25%, but were performed on different populations (16–20 year olds only, men only and the general population) [10], [11], [14], [17]. One of these studies was conducted in men in Washington (USA) with a similar age range to our study (18–24 years), and found an oral HPV prevalence of 7.5% [17]. The oral α-HPV prevalence in three Scandinavian studies has been lower (1.0%–2.8%), with the exceptions of one performed at a sexual health clinic for youths, where it was much higher [7], [13], [18], [19]. Reasons for the different HPV prevalences reported could be due to different sampling methods used (rinse with mouthwash or saline, using varying volumes, as well as using a cytobrush) and some differences in HPV detection methods used (different PCR primer pairs and methods for HPV typing (sequencing or arrays)). Aside from these methodological differences, there may also be true differences in HPV prevalence between the different populations investigated.

The most commonly detected HPV type in our study was HPV-18 detected in three of the seven positive samples. HPV-16, -67, -69 and -90 were isolated in one sample each. HPV-16 and -18 are both common high-risk HPV types, while HPV-67, -69 and -90 are less commonly reported types isolated from vaginal intraepithelial neoplasia and cervicovaginal cells [20]–[22]. The reason for HPV-67, -69 and -90 being less commonly reported could be because these types are not included in standard HPV assays; it does not necessarily mean that actual infection is less common.

We found that oral HPV infection was significantly associated with male gender. This has also been reported in the largest oral HPV infection population-based study to date, with 10.1% in men and 3.6% in women [10]. A Swedish study on adolescents found that the oral HPV prevalence was higher in young women (3.1%) compared to men (0.6%) [13], although this difference was not statistically significant. Male gender has previously been associated with higher HPV prevalence in HNSCCs [2].

Receiving oral sex was strongly associated with oral HPV infection in our population, but we found no associations with passionate kissing, giving oral sex or sexual intercourse (number or partners or frequency). Several other studies have found oral HPV infection to be associated with oral sex behaviours, number of partners as well as frequency [4], [14], [17], [23]. One multi-national study, of men from U.S., Brazil and Mexico, reported no association with oral sex and oral HPV prevalence [24].

Self-reported HIV diagnosis was found to be associated with oral HPV infection. HIV infection has previously been linked with increased odds ratio of prevalent oral HPV infection [25].

Some U.S studies have reported strong associations between marijuana use and oral HPV infection, both in healthy populations and HNSCC patients [4], [10], but we found no association with ever having used illicit drugs and oral HPV status.

About one third of the participants of our study had received one or more doses of the Gardasil® vaccine, and the great majority of these students were females (95/98). Only one study has evaluated the effect HPV vaccination has on oral HPV prevalence, using the bivalent HPV-16/-18 Cervarix® in a Costa Rican population and found that oral HPV prevalence was much lower in the vaccinated population after 4 years follow-up [26]. None of the oral HPV positive individuals in our sample were Gardasil® vaccinated. Four of the oral HPV positive students had infections with HPV-16 and -18, which potentially could have been prevented by HPV vaccination.

Strengths of the current study include the collection of detailed lifestyle and sexual behaviour information from all participants. Also, a sensitive technique was used for determining the presence of α-HPV DNA in the oral rinse samples. However, while the sample was relatively large, the prevalence of oral HPV was low, and our study was underpowered to fully explore associations with all possible explanatory factors. While few of the associations were statistically significant, the magnitude and direction of the associations are plausible with current knowledge, and should be examined in larger datasets or through meta-analysis. In addition, our sample was recruited from a single tertiary education institution, and thus while our findings cannot be generalised to all Australians in the same age group, we believe they are likely to reflect the experiences of those in similar settings.

In conclusion, we found that oral HPV prevalence was low in this sample of young Australians. Oral HPV infection was found to be associated with male gender, receiving oral sex and self-reported diagnosis of HIV. Given the low HPV DNA prevalence in this study, larger studies are required both in Australia and internationally to determine oral HPV infection in the general population.
